# Effects of 40 Hz Brain Stimulation Across Modalities: A Comparative Narrative Review

**DOI:** 10.3390/brainsci16080808

**Published:** 2026-07-30

**Authors:** Eugen Kvašňák

**Affiliations:** Third Faculty of Medicine, Charles University, 12000 Prague, Czech Republic; eugen.kvasnak@lf3.cuni.cz

**Keywords:** gamma oscillations, 40 Hz, non-invasive brain stimulation, transcranial alternating current stimulation (tACS), auditory steady-state response (ASSR), visual flicker, rTMS, multisensory stimulation, Alzheimer’s disease

## Abstract

Gamma-band oscillations centered around 40 Hz play an important role in cortical communication, and their disruption has been documented as a neurophysiological feature of several neurodegenerative and neuropsychiatric disorders. This narrative review synthesizes preclinical and early-phase clinical evidence for 40 Hz non-invasive brain stimulation across five delivery modalities: (1) auditory stimulation, which leverages the 40 Hz auditory steady-state response (ASSR) to probe parvalbumin-positive (PV^+^) interneuron circuits and serves as a validated neurophysiological biomarker in schizophrenia; (2) visual stimulation, using luminance or invisible spectral flicker to induce steady-state visually evoked potentials (SSVEPs) and, in animal models, to activate microglial phagocytosis; (3) transcranial alternating current stimulation (tACS), which delivers sinusoidal sub-threshold membrane polarization at gamma frequency, with preliminary case-series evidence suggesting tau burden reduction and EEG-based biomarker changes in Alzheimer’s disease; (4) repetitive transcranial magnetic stimulation (rTMS), offering focal cortical entrainment that, when combined with tACS in phase-synchronized protocols, produces sustained gamma enhancement in the dorsolateral prefrontal cortex; and (5) multisensory combined stimulation, which engages multiple convergent pathways and currently represents the approach with the most promising early translational signal, including cognitive stabilization and hippocampal volume preservation in small AD trials. While single-session entrainment does not reliably yield cognitive gains, multi-week applications have shown neurophysiological and preliminary biomarker-level changes in selected populations. It should be emphasized, however, that the human evidence base remains early-phase and largely derived from small, often uncontrolled studies; 40 Hz stimulation should accordingly be regarded as a biologically plausible and well-tolerated investigational approach rather than an established therapeutic intervention. Adequately powered, randomized, sham-controlled trials are required before clinical conclusions can be drawn.

## 1. Introduction

Gamma-band oscillations centered around 40 Hz represent one of the most studied frequency regimes in systems neuroscience. First characterized at the cellular level through the coordinated activity of fast-spiking, parvalbumin-positive (PV^+^) GABAergic interneurons driving rhythmic excitation/inhibition cycles in cortical networks, 40 Hz activity has been associated with processes of perception, attention, working memory, and large-scale cortical communication. The mutual influence among neuronal groups is thought to depend on their phase relationship at gamma frequencies: synchronized groups may exert stronger inter-area influence than desynchronized ones, a mechanism proposed to underlie the flexible routing of sensory information across cortex [1,2,3]

Gamma oscillations are generated by the interplay between pyramidal cells and PV^+^ interneurons in a circuit whose integrity depends on intact GABAergic signaling and NMDA-receptor function. Disruption of these circuits—through interneuron loss, GAD67/GAT-1 downregulation, amyloid/tau pathology, or dopaminergic dysregulation—produces characteristic reductions in gamma-band power and phase-locking that have been documented in Alzheimer’s disease (AD), schizophrenia, Parkinson’s disease (PD), and neurodevelopmental disorders.

The recognition that externally delivered 40 Hz stimulation might restore or augment these disrupted oscillations has driven a rapidly expanding research effort across at least five delivery modalities: (1) auditory stimulation via click trains or amplitude-modulated sounds evoking an auditory steady-state response (ASSR); (2) visual stimulation via 40 Hz luminance flicker or invisible spectral flicker; (3) transcranial electrical stimulation, primarily tACS; (4) transcranial magnetic stimulation (TMS/rTMS); and (5) multisensory combined protocols. A sixth modality—mechanical/vibrotactile stimulation—has begun to attract interest but remains at a substantially earlier stage.

### 1.1. Terminology and Levels of Evidence

To support consistent interpretation throughout this review, key terms are defined here. **Gamma entrainment** refers to the alignment of endogenous cortical oscillatory phase or power to the frequency of an external stimulus; it is an electrophysiological phenomenon measurable by EEG. **Synchronization** denotes the temporal coordination of neural firing within or between brain regions, which may or may not accompany entrainment as measured by scalp EEG. **Evoked response** (or steady-state evoked potential) refers to the time-locked, frequency-specific EEG response to a periodic sensory stimulus. **Target engagement** is used here strictly to mean demonstrable modulation of the neural target—confirmed by EEG, MEG, or neuroimaging—and is treated as a distinct level of evidence from **biomarker change** (e.g., altered CSF amyloid, MRI volumetry) and **clinical efficacy** (improvement in a pre-registered primary clinical outcome in a controlled trial). These four levels—mechanistic rationale, target engagement, biomarker modulation, and clinical efficacy—are treated throughout this review as distinct and require separate evaluation.

### 1.2. Review Methodology

This is a narrative review. Literature was identified through structured searches of PubMed, Scopus, and Google Scholar, covering publications up to June 2025. The primary search terms were: “40 Hz stimulation,” “gamma entrainment,” “auditory steady-state response,” “visual flicker Alzheimer,” “tACS gamma,” “rTMS 40 Hz,” and “multisensory gamma stimulation,” combined with disease terms including “Alzheimer’s disease,” “schizophrenia,” “Parkinson’s disease,” and “autism.” Reference lists of identified reviews and primary studies were hand-searched. Inclusion criteria: (1) human or animal studies reporting outcomes of 40 Hz (or 35–45 Hz) sensory, electrical, or magnetic stimulation; (2) publications in peer-reviewed journals with sufficient methodological detail to assess stimulation parameters and outcome measures; (3) case reports and feasibility studies were included when no controlled data existed for a given modality. Exclusion criteria: (1) studies using stimulation frequencies outside 35–45 Hz without direct 40 Hz comparison; (2) reviews and meta-analyses were used for background context but are not counted among the approximately 38 primary sources; (3) conference abstracts and unpublished data without peer-reviewed reporting. Preclinical studies were included when they directly informed the mechanistic rationale or helped interpret conflicting human findings. Evidence from non-AD conditions (schizophrenia, Parkinson’s disease, ASD, PTSD) is included where it provides mechanistic insight or supplies the only available data on a given modality, in keeping with the review’s primary comparative aim. No formal quality assessment or PRISMA screening was applied; the review is therefore subject to the limitations of narrative synthesis, including potential selection bias toward positive findings. Evidence is described alongside study design and sample size to allow the reader to calibrate confidence appropriately. Although no formal quality appraisal tool was applied, individual studies were evaluated against the following criteria: (1) study design (case series, single-arm pilot, registered Phase II/III RCT); (2) sample size and adequacy of statistical power; (3) presence and adequacy of sham or active control conditions; (4) independent replication by groups not involved in the original study; (5) duration of follow-up (acute/single-session versus multi-week sustained intervention); (6) use of objective biomarker endpoints alongside subjective or clinician-reported outcomes; and (7) overall methodological quality including blinding, randomization, and prospective registration. These criteria are noted throughout the text to support calibrated interpretation of the strength of individual findings.

The primary aim of this review is a comparative analysis of the five principal 40 Hz stimulation modalities—auditory, visual, electrical (tACS/tDCS), magnetic (rTMS), and multisensory combined—evaluated across four hierarchical evidence levels: neurophysiological target engagement, biomarker-level change, cognitive or functional outcome, and clinical feasibility. Secondary aims are to identify shared downstream pathways (microglial activation, glymphatic clearance, synaptic plasticity, and network synchrony), to characterize methodological considerations that limit cross-study comparisons, and to map the translational gradient from preclinical to early-phase human evidence. This review does not make clinical treatment recommendations; findings are presented to inform the design of adequately powered confirmatory trials.

## 2. Auditory Stimulation

### 2.1. Principles

The brain responds to periodic auditory stimuli by generating an oscillatory cortical response phase-locked to the stimulation frequency, termed the auditory steady-state response (ASSR). At 40 Hz, this response is particularly robust and has been mapped to both the primary auditory cortex and, through cortico-cortical projections, the prefrontal cortex and hippocampus. The 40 Hz ASSR is thought to be selectively generated by the same PV^+^ interneuron–pyramidal cell circuits that produce endogenous gamma oscillations, making it both a probe of gamma circuit integrity and a potential means of driving those circuits via sensory afferents. Importantly, ASSR amplitude and phase precision are modulated by arousal level [4], a factor relevant to protocol design and inter-study comparability.

### 2.2. Technique and Parameters

Stimuli are typically trains of clicks (25 ms inter-stimulus interval, yielding 40 Hz) or amplitude-modulated (AM) tones with a 40 Hz modulation rate, delivered binaurally or monaurally through headphones at 60–80 dB SPL. Binaural beats—presenting slightly offset frequencies to each ear to produce a 40 Hz beat percept—represent a softer variant used in sleep and relaxation applications. Experimental sessions range from several minutes to one hour; therapeutic protocols have used daily application over weeks to months. EEG recorded simultaneously measures ASSR power and inter-trial phase coherence (ITPC) as primary neurophysiological outcomes.

### 2.3. Populations Studied

Normative ASSR data have been established in healthy volunteers across the adult lifespan. Clinical populations in whom the ASSR deficit is well-characterized include: (a) *schizophrenia*, where reduced 40 Hz ASSR power and ITPC represent one of the most replicated neurophysiological findings [5,6,7]; (b) *22q11.2 deletion syndrome*, where ASSR attenuation in non-psychotic carriers correlates with negative symptoms [8]); (c) *Alzheimer’s disease*; and (d) *autism spectrum disorder*.

### 2.4. Effects Observed

**Target Engagement**. The ASSR is the primary neurophysiological outcome and provides direct evidence of target engagement. In schizophrenia, the characteristic deficit—reduced gamma power and ITPC at 40 Hz, with a paradoxical shift toward 20 Hz entrainment consistent with slowed inhibitory decay—is one of the most reliable biomarkers of the disorder and correlates with auditory hallucination severity [6,7]. In 22q11.2 deletion carriers, ASSR attenuation tracks premorbid circuit vulnerability [8].

**Cognitive Effects**. Wu et al. [9] found that single-session gamma audiovisual stimulation reliably induced EEG entrainment but did not enhance perception, attention, or short-term or long-term memory in healthy young participants. This dissociation between neurophysiological target engagement and behavioral improvement is an important observation: it indicates that entrainment, by itself, may be insufficient for acute cognitive benefit, at least over short timescales in healthy populations. In AD mouse models, chronic 40 Hz auditory stimulation reduces amyloid-beta and tau pathology and activates microglial phagocytosis with effects extending to the hippocampus; however, the translational relevance of these preclinical findings for human disease has not yet been established.

**Affective Effects**. Binaural 40 Hz beats have been associated with reduced self-reported anxiety and improved mood in small, largely uncontrolled studies. Effect sizes are modest and the evidence base limited.

**Sensory Effects**. The ASSR itself is the primary sensory neurophysiological outcome. Binaural beat responses at gamma frequency have been proposed to play roles in spatial hearing and sound-source segregation mediated by interhemispheric gamma synchrony [10].

### 2.5. Clinical Translation

The ASSR deficit is a well-validated target-engagement biomarker in schizophrenia. However, no adequately powered therapeutic entrainment trial in schizophrenia has been published, a notable gap given the mechanistic rationale. In AD, pilot studies have demonstrated tolerability and preliminary neurophysiological changes; larger sham-controlled trials are needed before claims about clinical efficacy can be made. ASSR recording is increasingly used as a surrogate endpoint for gamma circuit target engagement in clinical trials of both non-invasive stimulation and pharmacological interventions.

## 3. Visual Stimulation

### 3.1. Principle

Iaccarino et al. [11] demonstrated in 5xFAD mice that one hour of 40 Hz visual flicker (termed GENUS: Gamma ENtrainment Using Sensory Stimuli) reduced amyloid-beta levels by approximately 40–50% in the visual cortex, activated microglia into a phagocytic state, and enhanced microglial association with plaques. This influential preclinical finding motivated subsequent studies exploring propagation beyond the visual cortex and human applications. Henney et al. [12] extended the principle by demonstrating that invisible spectral flicker (ISF)—avoiding the perceptible luminance change of conventional flicker—can evoke steady-state gamma responses across the 36–44 Hz range.

An important challenge to this preclinical foundation was raised by Soula et al. [13], who reported, using multi-site silicon probe recording in visual cortex, entorhinal cortex, and hippocampus of APP/PS1 and 5xFAD mice, that 40 Hz flickering light did **not** engage native gamma oscillations in these regions, that hippocampal spike responses were weak, that mice avoided the flicker, and that no reliable changes in plaque count, microglia morphology, or amyloid-beta levels were observed. This finding is in direct tension with the Iaccarino report, and the discrepancy has not been resolved; methodological differences in recording approach, stimulation device, mouse line, and outcome measures likely contribute. This unresolved contradiction warrants cautious interpretation of the visual flicker preclinical literature.

### 3.2. Technique and Parameters

Visual stimulation is most commonly delivered as 40 Hz luminance flicker on an LED array, tablet screen, or purpose-built light panel, with a 50% duty cycle and typically one hour per session. ISF uses spectrally balanced light that modulates wavelength rather than luminance. Occipital EEG channels are used to verify SSVEP entrainment.

### 3.3. Populations Studied

Most rigorous data come from AD transgenic mouse models (5xFAD, APP/PS1, 3xTg). Human feasibility studies have been conducted in healthy older adults and individuals with mild cognitive impairment (MCI) or mild-to-moderate AD.

### 3.4. Effects Observed

**Target Engagement**. SSVEP magnitude and spatial extent of gamma power increase in the occipital cortex are robust indicators of visual target engagement. Propagation to frontal and hippocampal regions is debated and appears to depend on stimulation duration, individual factors, and recording methodology.

**Cognitive Effects**. In multiple AD mouse models, visual 40 Hz flicker has been associated with improved spatial memory and reduced pathological burden. In humans, pilot and feasibility studies generally report cognitive stabilization over weeks to months (measured by ADAS-Cog or MoCA), but sample sizes are small and controls are limited or absent in most published work. The single-session null result in healthy participants [9] confirms the dissociation between acute entrainment and cognitive improvement.

**Affective and Motor Effects**. Not systematically studied as primary outcomes in visual-only protocols. The observation that mice avoid high-intensity flicker raises a question about aversive properties relevant to long-term human adherence.

### 3.5. Clinical Translation

Evidence of hippocampal and entorhinal volume preservation over months is observed in some studies, but most of these data come from multisensory protocols (Section 5). SSVEP magnitude may predict individual capacity for gamma entrainment and could serve as a predictive biomarker for treatment response. Given the unresolved Soula et al. [13] challenge to the preclinical foundation, human findings should be interpreted independently of the mouse model literature until the mechanistic discrepancy is addressed.

## 4. Transcranial Electrical Stimulation

### 4.1. Principle

Transcranial alternating current stimulation (tACS) at 40 Hz delivers a sinusoidal current through scalp electrodes that, after transcranial attenuation through skull and cerebrospinal fluid, produces oscillating electric fields of approximately 0.1–0.5 V/m within the cortex, sufficient to produce rhythmic sub-threshold membrane polarization. The proposed mechanism is resonance-based entrainment: when the externally applied oscillation matches the natural frequency of an endogenous circuit, the circuit’s phase may be progressively aligned (phase-locking) and its power enhanced. This effect is state-dependent—it requires residual endogenous oscillatory capacity in the target network [14].

An important practical consideration is tolerability. Kvasnak and colleagues characterized perception and pain thresholds of tACS across frequencies and found that 40 Hz tACS has among the highest perception and pain thresholds of all tested frequencies—meaning it is the best-tolerated tACS frequency range in terms of cutaneous sensation [15,16]. Phosphene induction remains a confound for posterior electrode placements.

### 4.2. Technique and Parameters

Glinski et al. [17] demonstrated that phase-synchronized 40 Hz intermittent theta-burst stimulation (iTBS) combined with tACS produced the most stable and sustained enhancement (up to 2 h) of induced gamma oscillations in the dorsolateral prefrontal cortex (DLPFC) compared to either protocol alone, suggesting additive entrainment when magnetic and electrical modalities are combined.

### 4.3. Populations Studied

Healthy young adults, healthy older adults, patients with MCI and AD. Key populations include: participants in fluid intelligence training studies [18] and attention studies [19]; and AD patients in tele-supervised home-based tACS trials [20] and PET biomarker studies [21].

### 4.4. Effects Observed

**Target Engagement**. Phase-specific effects of tACS on SSVEP amplitude have been demonstrated by Fiene et al. [22], confirming that tACS can modulate visual cortex oscillatory responses in a phase-dependent and baseline-synchronization-dependent manner.

**Cognitive Effects**. The systematic review by Klink et al. [23] found that gamma-tACS broadly improved performance in auditory and visual perception tasks in healthy adults but did not consistently change executive function performance. Effects on working memory are mixed: some studies report enhancement of prefrontal–hippocampal connectivity; others [18] found no improvement in fluid intelligence with 40 Hz multifocal tACS combined with cognitive training. Hopfinger et al. [19] found a frequency-specific improvement in attentional disengagement (but not engagement) with 40 Hz (not 10 Hz) tACS in the right parietal cortex. In AD, the home-based HD-tACS pilot [20], reported memory improvement in all participants over 14 weeks, with an associated decrease in EEG theta/gamma ratio; however, global cognition (MoCA) did not improve significantly, and the absence of a sham control limits interpretation.

**Affective Effects**. The combination of gamma-tACS with other modalities for depression has been explored [24], though the primary frequency target for depression protocols is typically theta or alpha rather than 40 Hz.

**Motor Effects**. Motor cortex excitability studies have primarily used higher-frequency tACS (ripple range, 80–250 Hz) [25]. Indirect motor effects in PD have been explored in multisensory contexts (Section 5).

### 4.5. Clinical Translation

The most clinically relevant tACS finding is the AD biomarker case series by Dhaynaut et al. [21], in which 4 AD participants received daily 1 h 40 Hz bitemporal tACS for 4 weeks. PET imaging revealed tau burden reduction (>2%) in 3 of 4 patients in the targeted temporal lobe, particularly mesial structures. Amyloid plaque load did not change significantly. EEG showed increased gamma spectral power post-treatment. These preliminary data are hypothesis-generating; the *n* = 4 uncontrolled design does not permit efficacy conclusions. A 2025 perturbation-based tACS-EEG study [26] found positive correlations between 40 Hz-induced gamma power and cognitive status across 14 AD patients, supporting the utility of tACS-evoked gamma as a disease-severity biomarker. Broader clinical context and the need for controlled trials are discussed by McDermott et al. [27] and De Paolis et al. [28].

## 5. Transcranial Magnetic Stimulation

### 5.1. Principle

Repetitive transcranial magnetic stimulation (rTMS) delivers brief magnetic pulses through a scalp coil, inducing eddy currents in the cortex capable of depolarizing neurons. At 40 Hz, rTMS directly engages the gamma-frequency range and can entrain gamma oscillations in cortical circuits, though the focal nature of rTMS restricts spatial reach to the superficial cortex. TMS-EEG (concurrent high-density EEG recording during TMS) provides a direct readout of cortical natural oscillation frequency and gamma-band reactivity as a target-engagement biomarker.

Combined phase-synchronized iTBS with concurrent 40 Hz tACS produced the most stable long-lasting enhancement of gamma oscillations in the study by Glinski et al. [17], suggesting that magnetic and electrical modalities can act synergistically on shared circuits.

### 5.2. Technique and Parameters

Standard high-frequency rTMS uses figure-of-eight coils at 80–120% of resting motor threshold. Forty Hz rTMS trains are typically brief to avoid excessive heat and seizure risk. iTBS delivers bursts of three pulses at 50 Hz repeated at 5 Hz (theta), with the gamma-range component embedded in the burst structure. H-coil and deep TMS systems can reach deeper cortical and subcortical structures. After-effects of rTMS on MEP amplitude show complex time courses depending on train length [29].

### 5.3. Populations Studied

Healthy adults (for basic gamma entrainment studies), older adults and patients with MCI/AD (for therapeutic and biomarker studies), schizophrenia patients, and PTSD patients. The combined tACS-iTBS study by Glinski et al. [17] used healthy young adults.

### 5.4. Effects Observed

**Target Engagement**. Reduced gamma TMS-evoked potentials are observed in MCI and AD compared to controls, paralleling the ASSR deficit and supporting TMS-EEG as a cross-modal target-engagement tool.

**Cognitive Effects**. rTMS at gamma-range frequencies applied to DLPFC or temporal cortex has been associated with improvements in working memory and processing speed in older adults and patients with MCI in several open-label and controlled pilot studies. The combined iTBS + tACS protocol [17] demonstrated sustained gamma enhancement in DLPFC for up to 2 h, suggesting that rTMS-based entrainment can outlast the stimulation period—a potentially important window for post-stimulation cognitive training.

**Affective Effects**. rTMS at 10 Hz or 1 Hz over DLPFC has established RCT evidence for major depressive disorder. The specific contribution of 40 Hz rTMS protocols to affective outcomes is less studied; the review by Antal et al. [30] notes that TMS and tES converge in their ability to enhance cognition, but that the parameter space is large and outcomes variable.

**Motor Effects**. Motor cortex rTMS has established clinical applications in stroke rehabilitation, though not specifically at 40 Hz. Gamma stimulation effects in PD are primarily explored in multisensory contexts.

**Sensory Effects**. TMS-EEG directly measures natural oscillation frequencies and gamma-band power in response to magnetic perturbation, providing a non-confounded readout of cortical excitability at the target frequency.

### 5.5. Clinical Translation

The most directly relevant finding is from Glinski et al. [17], which demonstrates that combined 40 Hz iTBS and tACS produces significant and sustained gamma power enhancement in DLPFC. The biomarker utility of gamma TMS-EEG in AD and the therapeutic potential of gamma-range rTMS are active areas of investigation. Both Antal et al. [30] and Hunold et al. [31] highlight that inter-individual anatomical variability substantially limits reproducibility of rTMS effects, motivating individualized dose planning.

## 6. Multisensory Combined Stimulation

### 6.1. Principle

Synchronizing two or more modalities of 40 Hz stimulation simultaneously engages multiple sensory processing streams and their convergence zones, potentially producing greater and more widespread network entrainment than any single modality alone. The most studied combination is simultaneous auditory and visual 40 Hz stimulation (audiovisual GENUS). Preclinically, this has shown additive or synergistic effects on amyloid clearance, microglial activation, and synaptic preservation beyond what either unimodal stimulation achieves. An additional proposed mechanism involves the glymphatic system: rhythmic neural activity at 40 Hz may drive CSF pulsatility and accelerate clearance of amyloid and tau, complementing direct microglial phagocytosis. Combined audiovisual stimulation may also engage the default mode network and hippocampal circuits more effectively than unimodal stimulation.

### 6.2. Technique and Parameters

Simultaneous delivery of 40 Hz visual flicker (LED goggles or panel display) and 40 Hz auditory click train or AM sound through headphones, for 30–60 min daily. Trials in AD have extended to 3 and 12 months. Dedicated wearable devices combining both modalities have been developed for home-based therapeutic use.

### 6.3. Populations Studied

Patients with AD and MCI in feasibility and proof-of-concept trials; PD patients in preliminary motor studies; and healthy older adults in short-term assessments.

### 6.4. Effects Observed

**Target Engagement**. Combined stimulation produces larger and more spatially distributed increases in gamma power than either modality alone, as assessed by EEG, with more consistent hippocampal and parahippocampal involvement.

**Cognitive Effects**. The most encouraging human evidence in the 40 Hz stimulation field comes from combined audiovisual protocols. Feasibility data demonstrate tolerability and adherence exceeding 90% in motivated samples over months. Controlled studies have reported cognitive stabilization—operationalized as attenuation of decline on ADAS-Cog—over 3-month and 6-month follow-up periods. A 3-month randomized pilot of daily 40 Hz audiovisual stimulation (GENUS) reported hippocampal volume stabilization and improved delayed recall compared with sham [32]. A 6-month randomized trial (OVERTURE) demonstrated reduced entorhinal white matter atrophy and attenuated whole-brain volume loss in the active arm relative to sham [33,34]. These are promising findings, but they derive from small trials conducted by a limited number of research groups; independent replication and phase III confirmation are needed.

**Motor Effects**. In PD, a preliminary trial of combined audiovisual 40 Hz stimulation reported improvements in gait speed and non-motor symptoms over a short treatment period, likely reflecting entrainment of basal ganglia–cortical gamma circuits.

**Affective Effects**. Not systematically addressed as primary outcomes in published multisensory trials.

### 6.5. Clinical Translation

Multisensory 40 Hz stimulation currently represents the approach with the most encouraging early-translational clinical signal for AD, while the evidence base remains preliminary and restricted to small, non-Phase-III studies, combining acceptable safety and tolerability with preliminary biomarker and cognitive outcome data extending to 6 months [32,33,34]. Whether benefits are durable beyond 6 months, and how outcomes compare with approved anti-amyloid pharmacotherapy, remain unanswered questions. Head-to-head comparisons between audiovisual and unimodal protocols in adequately powered trials have not been published.

## 7. Biomarkers of 40 Hz Stimulation

### 7.1. Distinguishing Levels of Evidence

A structured biomarker framework is essential for interpreting evidence across modalities. Three categories of biomarkers are relevant:

1. **Target-engagement biomarkers**: These confirm that the stimulation is reaching and modulating the intended neural target. They include EEG gamma power and ITPC (during and after stimulation), SSVEP amplitude and spatial distribution (visual), ASSR power and phase coherence (auditory), and TMS-EEG evoked gamma potentials.
2. **Disease-progression biomarkers**: These reflect the underlying disease state and may provide evidence of disease modification if changed following treatment. They include structural MRI (hippocampal and entorhinal volume), amyloid PET and tau PET, and CSF concentrations of Aβ42, p-tau181, and t-tau.
3. **Treatment-response and emerging plasma biomarkers**: Plasma p-tau181 and p-tau217, neurofilament light chain (NfL), and glial fibrillary acidic protein (GFAP) are increasingly validated as disease-severity and progression markers in AD; longitudinal changes in these markers following sustained stimulation protocols represent a priority outcome for future trials. Sleep architecture measures (PSG; slow-wave and REM sleep, sleep spindles) are relevant because disrupted sleep is a feature of AD pathogenesis and the glymphatic clearance mechanism may involve sleep-dependent CSF flow.

### 7.2. Methodological Considerations for Biomarker Measurement

**EEG artifact control**: tACS at the stimulation frequency introduces large electrode artifacts at 40 Hz and harmonics; online filtering and interleaved or perturbation-based paradigms (as in [26]) are required to recover genuine gamma responses. Phase-locking metrics (ITPC) are less susceptible to signal-to-noise confounds than spectral power measures and should be preferred as primary EEG outcomes.

**MRI/PET pipelines**: Hippocampal volumetry requires manual or validated automated segmentation (e.g., FreeSurfer) with registered longitudinal scans; standardized amyloid and tau PET centiloid conversion improves cross-study comparability.

**CSF and plasma variability**: Pre-analytical variability (time from lumbar puncture to centrifugation, freeze–thaw cycles) substantially affects CSF Aβ and tau measurements; plasma p-tau and NfL require Simoa or equivalent ultra-sensitive platforms. Biomarker changes in case series and small trials must be interpreted with awareness of these sources of variability.

### 7.3. Current Biomarker Findings by Modality

– *Auditory*: ASSR power and ITPC deficits in schizophrenia are well replicated; their use as target-engagement biomarkers in stimulation trials is growing. No disease-progression biomarker changes following auditory-only 40 Hz stimulation have been reported in humans.
– *Visual*: SSVEP provides reliable target-engagement evidence; preclinical disease-progression biomarker changes (amyloid, microglia) are substantial but contested [13]. No robust human disease-progression biomarker data exist for visual-only protocols.
– *tACS*: Dhaynaut et al. [21] provide preliminary PET tau reduction data; Palmisano et al. [26] demonstrate correlations between tACS-induced gamma and clinical severity. Both studies are small and uncontrolled.
– *rTMS*: TMS-EEG gamma reduction in MCI/AD is a valuable target-engagement biomarker; no disease-progression biomarker data from 40 Hz rTMS trials are available.
– Multisensory: Reduced entorhinal white matter atrophy and whole-brain volume preservation represent the most extensively reported disease-progression biomarker signals across modalities to date, supported by data from the 6-month OVERTURE trial [33,34]. Preliminary hippocampal stabilization and cognitive improvement data are also available from the 3-month GENUS pilot [32]. These findings require independent replication.

## 8. Safety and Clinical Implementation

### 8.1. General Tolerability

Across published studies, 40 Hz non-invasive stimulation is generally well tolerated. Adverse events are predominantly mild and transient. Sensory modalities (auditory and visual) carry the lowest systemic risk profile. tACS at 40 Hz is the best-tolerated tACS frequency in terms of cutaneous sensation thresholds [15,16]. rTMS has established safety guidelines [10] and is well tolerated at clinical intensities when standard protocols are followed.

### 8.2. Modality-Specific Risks

**Visual flicker**: Photic stimulation at any frequency carries a low but non-zero risk of triggering seizures in individuals with photosensitive epilepsy. Standard screening for personal and family history of seizures is required. Screen brightness, room luminance, and session duration should be controlled. The potential for discomfort or aversion at high flicker intensities (as observed in mice) should be monitored in human trials.

**tACS**: Phosphenes can be induced by posterior electrode placements due to retinal co-stimulation and may affect blinding. Skin irritation at electrode sites is common. Participants with implanted ferromagnetic or electrically active devices (pacemakers, cochlear implants, deep brain stimulators) should be excluded. Home-based tACS protocols [20] require tele-supervision and clear safety protocols for adverse event identification and reporting.

**rTMS**: Standard contraindications include implanted metal or electronic devices in or near the skull, history of seizure, and current use of proconvulsant medications. Auditory protection is required due to coil click noise. Seizure risk, although low with standard protocols, necessitates trained personnel and emergency preparedness.

**Multisensory combined protocols**: Risks are additive across modalities. Prolonged daily use over weeks to months requires structured safety monitoring, standardized adverse event recording, and periodic clinical review.

### 8.3. Practical Implementation

Home-based delivery of sensory modalities and remote-supervised tACS is feasible and supports adherence, which is critical for conditions requiring chronic daily treatment. The low cost of LED flicker and headphone delivery contrasts with the clinic-based requirements and cost of rTMS, giving sensory modalities a clear implementation advantage at the population level. Adherence in published sensory stimulation trials exceeds 90% in motivated samples, but this likely reflects selection bias; community-level adherence data are lacking.

## 9. Comparative Analysis

### 9.1. Summary Table of 40 Hz Stimulation Modalities

Table 1 is the summary table of 40 Hz stimulation modalities.

### 9.2. Summary of Key Human Studies by Modality

Table 2 is the summary of key human studies by modality.

### 9.3. Key Findings from Comparative Analysis

Several patterns emerge across modalities. First, 40 Hz is a neurophysiologically relevant frequency: it corresponds to the resonant frequency of PV^+^ interneuron circuits, produces the most robust steady-state responses in auditory and visual cortex, and is the best-tolerated tACS frequency. These observations are robust across independent laboratories. Second, target engagement and clinical benefit are dissociable: single-session entrainment does not reliably produce cognitive gains even in healthy populations [9]; therapeutic effects, where present, appear to require sustained daily application over weeks to months. Third, spatial propagation is a key constraint: sensory modalities engage networks through anatomical pathways but may not reliably reach deep structures (hippocampus, entorhinal cortex) that are critical for AD pathology modification; electrical and magnetic stimulation can target these regions more directly at the cost of device complexity. Fourth, the multisensory approach partially circumvents the single-modality spatial limitation, which may help explain the relatively stronger translational signal observed so far [24,32,35]. Fifth, there is a meaningful dissociation within electrical stimulation: tACS at 40 Hz imposes oscillatory entrainment, while tDCS enhances excitability; their combination with sensory modalities may be synergistic [36].

## 10. Discussion

### 10.1. Mechanisms of Action

**Figure 1:**
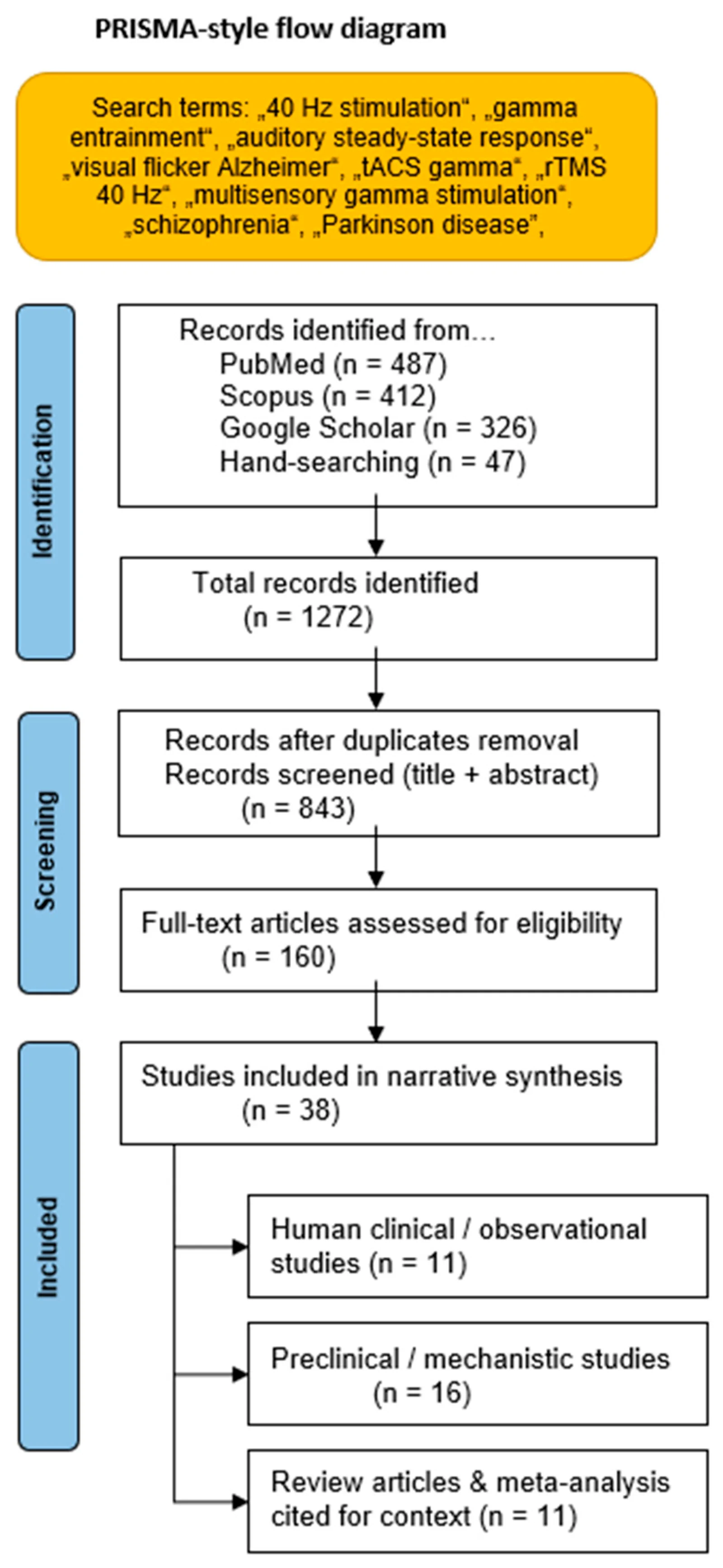
PRISMA-style flow diagram of the literature search and selection process. Records were identified through database searches (PubMed, *n* = 487; Scopus, *n* = 412; Google Scholar, *n* = 326; total *n* = 1225), with an additional 47 records from hand-searching of reference lists. After deduplication, 843 records were screened by title and abstract; 683 were excluded. Of 160 full-text articles assessed for eligibility, 122 were excluded (wrong frequency, no neurophysiological outcome, or duplicate reporting), leaving 38 studies included in the final narrative synthesis. Record counts are illustrative; exact numbers were not recorded prospectively.

Across all five modalities (Figure 1), the core hypothesis is that driving neural circuits at 40 Hz can restore the phase and power of endogenous gamma oscillations disrupted in neurological disease. The proposed downstream consequences are multiple: (1) microglial phagocytic activity may be upregulated, accelerating clearance of amyloid-beta and tau aggregates—this is the best-supported preclinical mechanism [11,34]; (2) the glymphatic system’s pulsatility may be influenced by faster oscillations, enhancing interstitial metabolite clearance—this remains the most speculative mechanism; importantly, there is currently no direct human evidence that 40 Hz stimulation enhances glymphatic clearance in humans, with the proposed mechanism supported only by indirect preclinical and CSF flow data; (3) synaptic strengthening via spike-timing-dependent plasticity may be facilitated by coherent gamma-frequency pre- and postsynaptic activity; (4) large-scale network communication may be enhanced by phase alignment between distant cortical areas [1,2], supporting cognitive processes dependent on prefrontal–hippocampal synchrony; and (5) in PD, restored basal ganglia–cortical gamma synchrony may improve movement timing and gait rhythm.

The evidence assigns the greatest mechanistic certainty to the microglial pathway [11,33] and to network-level entrainment (human EEG/MEG). The glymphatic pathway remains speculative; critically, there is currently no direct human evidence demonstrating that 40 Hz stimulation enhances glymphatic clearance in humans, and this mechanistic claim should not be presented with a level of certainty that is not yet warranted. Synaptic plasticity and vascular effects are biologically plausible but less directly demonstrated. Notably, one study reported that 40 Hz visual flicker did not produce reliable entrainment or amyloid reduction in an AD mouse model under rigorously blinded conditions [13], representing an important contradicting result that tempers the overall mechanistic confidence in the visual modality.

**Figure 2:**
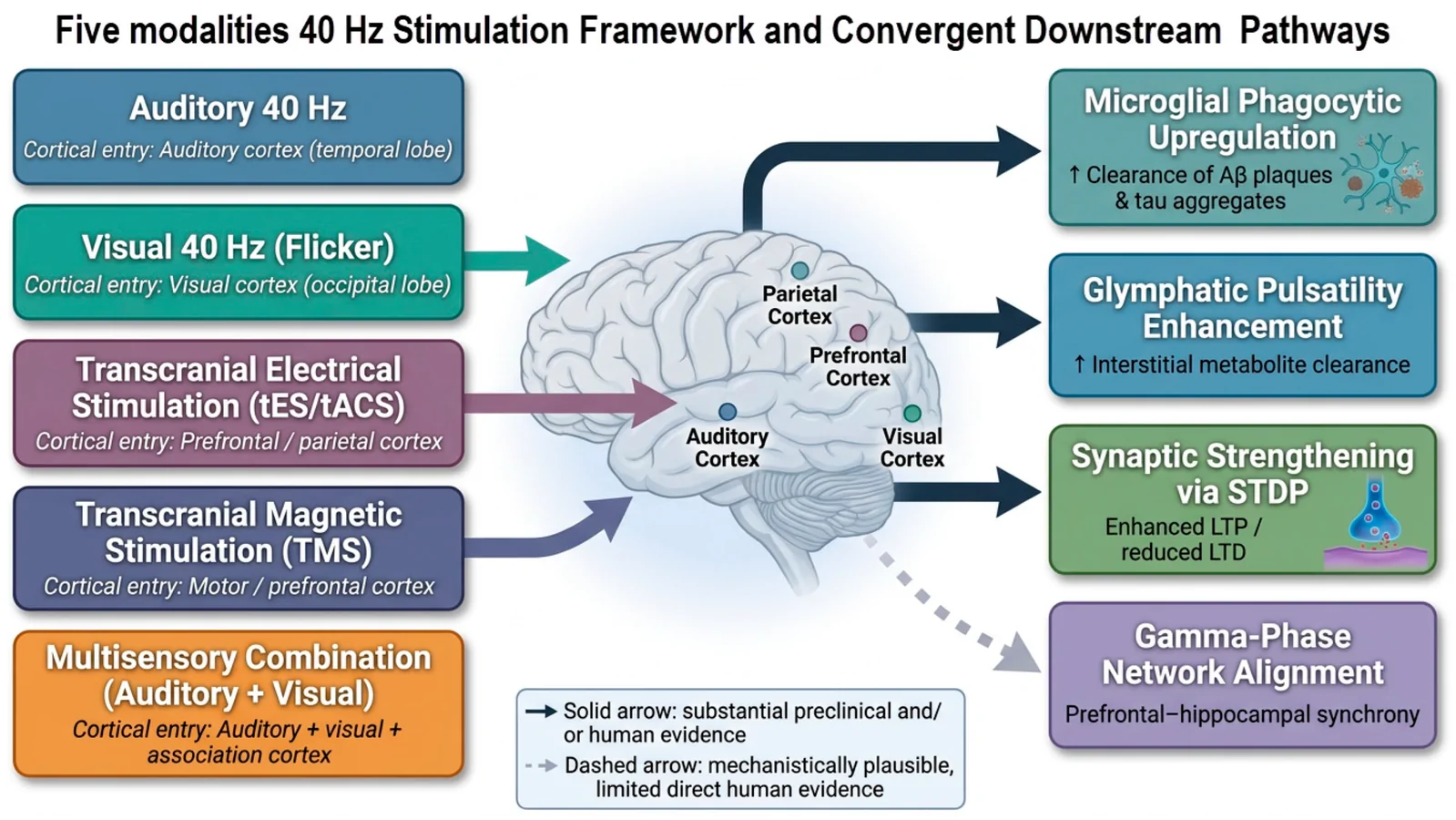
Summary schematic of five-modality 40 Hz stimulation framework and convergent downstream pathways. Each sensory modality (auditory, visual, transcranial electrical stimulation, transcranial magnetic stimulation, and multisensory combination) is mapped to its primary anatomical targets and cortical entry point. Arrows indicate convergence on four shared downstream pathways: (1) microglial phagocytic upregulation accelerating clearance of amyloid-beta and tau aggregates; (2) glymphatic system pulsatility enhancement facilitating interstitial metabolite clearance; (3) synaptic strengthening via spike-timing-dependent plasticity; and (4) large-scale network-level gamma-phase alignment supporting prefrontal–hippocampal synchrony. Evidence certainty is indicated by arrow weight: solid = substantial preclinical and/or human evidence; dashed = mechanistically plausible, limited direct human evidence.

The summary schematic of five-modality 40 Hz stimulation framework and convergent downstream pathways is shown in Figure 2.

### 10.2. Disease-Specific Considerations

**Alzheimer’s disease**: The most extensive translational evidence to date is in AD, with multisensory protocols showing the most consistent neurophysiological and preliminary biomarker signals. No Phase III trials with regulatory-grade power have been completed. The ASSR as a circuit-integrity biomarker and tACS-evoked gamma as a disease-severity biomarker both merit further validation in AD cohorts.

**Schizophrenia**: The ASSR deficit is the most validated neurophysiological biomarker in the disorder, yet therapeutic 40 Hz entrainment trials in schizophrenia are conspicuously absent—a gap given the strong mechanistic rationale. It is important to recognize that the therapeutic objective and expected clinical outcomes of 40 Hz entrainment in schizophrenia differ fundamentally from those in AD: the primary goal is to restore deficient gamma circuit output—not to clear amyloid or tau aggregates. ASSR amplitude and phase-locking serve as established biomarkers of cortical interneuron integrity in schizophrenia, and their normalization would be the appropriate primary neurophysiological endpoint in a gamma entrainment trial in this population, alongside cognitive composite measures. Dedicated trial designs with schizophrenia-specific outcome frameworks are required to advance this application.

**Parkinson’s disease, ASD, and PTSD**: These represent plausible but early-stage targets. PD has preliminary motor feasibility data; ASD and PTSD remain largely at the level of mechanistic hypothesis. Importantly, the disease-specific mechanisms, therapeutic objectives, and expected clinical outcomes differ substantially across these three conditions. In Parkinson’s disease, the primary neural target is basal ganglia–cortical gamma-band synchrony, which is disrupted during rest and movement; expected clinical outcomes center on motor improvements (gait, tremor, bradykinesia) rather than the cognitive or biomarker endpoints relevant to AD. In ASD, gamma oscillation abnormalities have been documented in auditory ASSR studies and may reflect interneuron-circuit dysfunction; however, the expected direction and magnitude of therapeutic response and the most meaningful outcome measures remain poorly defined. In PTSD, the rationale involves prefrontal–hippocampal connectivity and fear-memory consolidation processes, but no empirical 40 Hz entrainment trials have yet been conducted in this population; the therapeutic application remains at the level of mechanistic hypothesis. These distinctions require that evidence from each condition be evaluated independently rather than extrapolated from AD findings.

### 10.3. Methodological Considerations

Several issues recurrently limit interpretation. (1) **Sham controls** are difficult: participants can often detect active sensory stimulation, and tACS produces cutaneous sensations. (2) **EEG artifact** from tACS at the stimulation frequency requires careful filtering and/or interleaved/perturbation-based protocols. (3) **Individual variability in peak gamma frequency** (typically 35–45 Hz) means fixed 40 Hz may not be optimal for all participants; personalized frequency tuning based on ASSR peak could improve entrainment [4]. (4) **Inter-individual anatomical variability** produces large differences in the cortical electric field delivered by nominally identical tACS montages [15,31], motivating individualized current flow modeling. (5) **Closed-loop protocols** improve entrainment over open-loop but add technical complexity [14,37]. (6) The **Iaccarino/Soula contradiction** on visual flicker in AD mouse models has not been resolved and represents an active methodological debate. (7) A central and as yet unresolved question is whether electrophysiological target engagement is causally responsible for long-term clinical benefit: while several studies demonstrate successful gamma entrainment, it has not been established that restoring gamma oscillations is itself the mechanism driving disease modification or sustained cognitive improvement. The pathway from oscillatory entrainment to clinically meaningful benefit remains hypothetical and represents one of the most important open questions in the field; future trials should include designs capable of distinguishing true disease-modifying effects from symptomatic or nonspecific benefits.

A recurring methodological question concerns frequency specificity: whether 40 Hz is uniquely effective or whether adjacent frequencies would produce comparable outcomes. Direct evidence from tACS studies comparing 10 Hz and 40 Hz stimulation indicates qualitatively different cognitive and physiological effects [19,23], and preclinical studies targeting 40 Hz gamma consistently demonstrate superior amyloid and tau reduction compared with 20 Hz or 80 Hz controls [12]. The selectivity of 40 Hz is consistent with the resonant frequency of PV^+^ interneuron circuits, whose dysfunction is central to AD pathophysiology. Nevertheless, peak individual gamma frequencies vary between approximately 35 and 45 Hz across healthy adults [34], meaning that a fixed 40 Hz protocol may be suboptimal for a portion of participants. Future studies should incorporate individual ASSR-based frequency personalization to maximize entrainment fidelity and reduce inter-subject variability in outcomes.

### 10.4. Limitations of the Evidence Base

Most human trials are underpowered (*n* < 30), of short duration (<3 months), and lack adequately matched active control conditions. The concentration of multisensory AD trial evidence in a small number of research groups limits independent replication. Electrical stimulation trials use heterogeneous montages, intensities, and outcome measures that resist meta-analysis. The relationship between the magnitude of acute EEG entrainment and long-term clinical benefit has not been established. Long-term safety beyond 6 months has not been characterized. Populations with epilepsy, severe cardiac disease, or implanted devices have been systematically excluded, limiting generalizability. Publication bias represents an additional important limitation of the current evidence base: given that this field is still relatively small and largely composed of positive pilot studies concentrated in a limited number of research groups, the probability that neutral or negative findings remain unpublished cannot be excluded. The available evidence should therefore be interpreted with explicit acknowledgment that the published literature may overrepresent positive results, and the apparent consistency of positive signals across early-phase studies may partly reflect selective reporting rather than the true effect size.

### 10.5. Future Directions

Priority areas for future research include: (1) adequately powered, Phase III trials of multisensory 40 Hz stimulation in AD with pre-registered endpoints, including biomarker-defined subgroups; (2) systematic head-to-head comparisons between modalities in matched populations; (3) personalized frequency tuning and closed-loop delivery to improve entrainment efficiency; (4) clarification of the Soula vs. Iaccarino discrepancy using standardized recording and stimulation protocols; (5) therapeutic entrainment trials in schizophrenia and ASD, where the ASSR biomarker rationale is strongest; (6) development of multivariate predictive biomarkers (baseline ASSR, resting EEG gamma power, APOE genotype, plasma NfL/GFAP) for treatment response; and (7) combination strategies pairing gamma entrainment with pharmacotherapy (cholinesterase inhibitors, anti-amyloid antibodies) or cognitive training to exploit complementary mechanisms.

## 11. Conclusions

Preliminary convergent signals from multiple independent laboratories now indicate that externally delivered 40 Hz stimulation—across auditory, visual, electrical, and magnetic modalities—can produce measurable electrophysiological target engagement in humans and, in animal models, modulate microglial phagocytic activity and synaptic function. In humans, preliminary neurophysiological and, in selected populations, biomarker-level effects have been demonstrated. The multisensory audiovisual approach currently carries the most encouraging early translational signal for Alzheimer’s disease: the ASSR remains a well-validated target-engagement biomarker of gamma circuit integrity across neuropsychiatric disorders; tACS at 40 Hz provides a targeted, home-deployable approach with intriguing preliminary biomarker data; and the combination of rTMS with tACS in phase-locked protocols opens new optimization possibilities.

However, several important caveats must be emphasized. The human evidence base is predominantly early-phase, derived from small and often uncontrolled studies, and concentrated in a limited number of research programs. The distinction between electrophysiological target engagement and clinically meaningful disease modification has not yet been bridged by adequately powered, independently replicated trials. Forty Hz stimulation should therefore be regarded as a promising investigational approach with sound—though not yet definitive—mechanistic rationale, not as an established therapy. Executing the rigorous clinical trials needed to evaluate therapeutic efficacy remains the critical next step for the field.

## Figures and Tables

**Table 1:** Comparative overview of 40 Hz stimulation modalities.

Feature	Auditory	Visual	tACS	rTMS	Multisensory
Primary mechanism	ASSR entrainment	SSVEP entrainment	Direct oscillatory entrainment	Focal cortical depolarization	Convergent pathway entrainment
Target engagement biomarker	ASSR power/ITPC	SSVEP amplitude	EEG gamma power	TMS-EEG gamma	EEG gamma (distributed)
Spatial reach	Auditory cortex → PFC/Hippocampus	Occipital → (debated) frontal/hippocampal	Depends on montage	Focal (superficial cortex)	Multi-region (convergence zones)
Disease-progression biomarker data	None in humans	None in humans (robust)	Tau PET (*n* = 4)	None	MRI volume preservation (entorhinal white matter atrophy, whole-brain volume loss) [33,34]
Strongest clinical evidence	ASSR in schizophrenia (biomarker, not treatment)	AD feasibility	AD pilot/case series	Gamma enhancement (healthy)	AD proof-of-concept: hippocampal stabilization and improved recall (GENUS, 3 months [32]); reduced entorhinal WM atrophy and whole-brain volume loss (OVERTURE, 6 months [33,34])
Home-based feasibility	Yes	Yes	Yes (tele-supervised)	No	Yes
Tolerability profile	Very good	Good (photosensitivity caveat)	Excellent at 40 Hz	Moderate (clinical supervision required)	Good

**Table 2:** Summary of key human clinical findings by modality (Note: biomarker investigations—including target-engagement and disease-progression biomarker studies—and therapeutic intervention trials are distinguished within this table, as they represent fundamentally different levels of evidence that should not be directly compared).

Study	Modality	Design	N	Follow-Up Duration	Control	Primary Outcome	Biomarker Measures	Key Finding	Limitations	Evidence Level
Kwon et al., 1999 [5]	Auditory	Case-control	15 + 15	Single session	Healthy controls	ASSR power	EEG (ASSR power/ITPC)	Reduced 40 Hz ASSR in schizophrenia	Small N, single site	Replicated biomarker
Hirano et al., 2015 [7]	Auditory	Case-control	90 + 90	Cross-sectional	Healthy controls	Spontaneous gamma	EEG (spontaneous gamma)	Reduced gamma correlates with symptoms	Cross-sectional	Replicated biomarker
Wu et al., 2025 [9]	Audiovisual	Within-subject RCT	60	Single session	Sham	Cognitive performance	EEG (entrainment); cognitive tests	Entrainment without cognitive gain	Healthy young adults; single session	Null RCT
Dhaynaut et al., 2022 [21]	tACS	Open-label case series	4	4 weeks	None	Tau PET	Tau PET	Tau reduction in 3/4 patients	*n* = 4, no control	Pilot/hypothesis-generating
Cappon et al., 2023 [20]	tACS	Feasibility pilot	8	Not reported	None	Memory Index Score	Memory Index Score	Memory improvement in all patients	*n* = 8, no sham control	Pilot
Palmisano et al., 2025 [26]	tACS-EEG	Cross-sectional	14	Cross-sectional	None	Gamma induction	EEG (gamma induction)	Correlation with clinical severity	No intervention; cross-sectional	Biomarker association
Glinski et al., 2025 [17]	rTMS + tACS	Within-subject RCT	24	Single session	Sham	Gamma power (EEG)	EEG (gamma power)	Combined protocol: longest-lasting gamma enhancement	Healthy young adults; single session	RCT (target engagement)
Hopfinger et al., 2017 [19]	tACS	Crossover RCT	24	Single session	10 Hz and sham	Attention task	Attention task performance	40 Hz-specific attentional disengagement improvements	Healthy adults	RCT (cognitive)
Chan et al., 2022 [24]	Audiovisual (GENUS)	Randomized pilot (single-blind)	12	3 months	Sham	Hippocampal volume; cognition	MRI (hippocampal volume); EEG (entrainment); cognitive tests	Hippocampal stabilization; improved delayed recall; reduced ventricular dilation	Small N; single center; limited blinding	Pilot RCT
Hempel et al., 2023 [35]	Audiovisual (OVERTURE)	Randomized controlled (6 months)	Not reported	6 months	Sham	White matter volume (MRI)	MRI (white matter volume; myelin content)	Reduced entorhinal white matter atrophy; reduced myelin content loss	Post-hoc analysis; sample size not fully reported	RCT sub-analysis
Hajós et al., 2022 [32]	Audiovisual (OVERTURE)	Randomized controlled (6 months)	Not reported	6 months	Sham	Whole-brain volume (MRI)	MRI (whole-brain volume); ADL	Reduced whole-brain and occipital lobe atrophy; maintained ADL	Conference abstract; limited methodological detail	RCT (abstract)

## Data Availability

No new data were created or analyzed in this study.
